# Supplementary material for: A machine learning approach to managing game bird introductions
Source: PeerJ. 2025 Nov 4;13:e20291. doi: 10.7717/peerj.20291 (PMC12593725; doi:10.7717/peerj.20291)
Supplement: Supplemental Information 1 [file peerj-13-20291-s001.zip › Data/cookbook.pdf]

# A machine learning approach to managing game bird introductions

**Austin M. Smith[1,2] , Wendell P. Cropper, Jr.[3], Michael P. Moulton[1]**

[1]Department of Wildlife Ecology and Conservation, University of Florida, Gainesville, FL, 32611

[2]School of Natural Resource and Environment, University of Florida, PO Box 116455, Gainesville, FL, 32611

[3]School of Forest, Fisheries, and Geomatics Sciences, University of Florida, Gainesville, FL, 32611

## Codebook for Chukar CSV Files

This document describes the contents of two datasets used in the study, detailing chukar partridge (*Alectoris chukar*) introduction, presence, and harvest records across counties in Washington and Oregon.

## Dataset 1:

## `Galbreath_Moreland_1953_counties.csv`

| Column | Type / Values | Description |
|---|---|---|
| `County` | Character | Name of the county in Washington State. |
| `Recorded` | 1 = Yes<br>0 = No | Whether a Chukar record was documented in the county. |
| `Result` | 1 = Successful<br>0 = Unsure<br>-1 = Failed<br>-2 = No data | Overall outcome of introduction status in the county. |
| `R1938–R1951` | 1 = Recorded<br>0 = Not recorded | Annual record of Chukar release or observation from 1938 to 1951. |
| `Harvest` | 1 = Yes<br>0 = No | Whether Chukar harvest was reported in the county, based on Washington Department of Fish and Wildlife data (2014–2023). |

## Dataset 2: `Oregon_DFW_harvest_counties.csv`

| Column | Type / Values | Description |
|---|---|---|
| `County` | Character | Name of the county in Oregon State. |
| `Unit` | Integer (1–7) | Classification of wildlife management zones defined by the Oregon Department of Fish and Wildlife. |
| `Harvest` | 1 = Yes<br>0 = No | Whether Chukar harvest was reported in the county, based on Oregon Department of Fish and Wildlife data (2014–2023). |

## Notes

- All files are UTF-8 encoded CSVs with headers.
- No missing values are encoded as `NA`; zeros indicate absence or non-record.
- The `Result` column in the Washington dataset reflects qualitative assessments of introduction success based on historical records decribed in Galbreath & Moreland (1953).

## References


- Galbreath DS, and Moreland R. 1953. The chukar partridge in Washington: Washington State Game Department.
- Oregon Department of Fish and Wildlife. 2014-2023. Upland birds harvest information. Available at https://myodfw.com/articles/upland-birds-harvest-information (accessed 24 April 2025).
- Washington Department of Fish and Wildlife. 2014-2023. Game harvest reports and statistics. Available at https://wdfw.wa.gov/hunting/management/game-harvest (accessed April 24 2025).
