# Supplementary material for: A machine learning approach to managing game bird introductions
Source: PeerJ. 2025 Nov 4;13:e20291. doi: 10.7717/peerj.20291 (PMC12593725; doi:10.7717/peerj.20291)
Supplement: Supplemental Information 2 [file peerj-13-20291-s002.pdf]

Supplementary Table S1:

Hunting harvest statistics for chukar partridge (*Alectoris chukar*) in Washington State (2014–2023).

County	2014	2015	2016	2017	2018	2019	2020	2021	2022	2023
Adams	24	269	37	0	99	28	158	11	23	113
Asotin	1212	1630	1522	1072	2433	1714	4248	1295	569	1299
Benton	54	70	0	220	574	417	34	38	95	157
Chelan	1081	3378	2418	3177	3327	3954	3373	1929	805	676
Clallam	0	0	0	0	0	0	0	0	0	0
Clark	0	0	0	0	0	0	0	0	0	0
Columbia	280	0	0	52	209	0	24	0	8	9
Cowlitz	0	0	0	0	0	0	0	0	0	0
Douglas	702	1260	924	1248	1409	717	1487	416	824	1914
Ferry	0	0	0	0	0	0	0	0	0	0
Franklin	19	0	0	13	0	9	0	22	18	12
Garfield	245	195	270	174	367	304	48		44	32
Grant	212	1019	467	1498	883	669	533	781	328	82
Grays Harbor	0	0	0	0	0	0	0	0	0	0
Island	0	0	0	0	0	0	0	0	0	0
Jefferson	0	0	0	0	0	0	0	0	0	0
King	0	0	0	0	0	0	0	0	0	0
Kitsap	0	0	0	0	0	0	0	0	0	0
Kittitas	896	870	1426	1584	2207	2276	803	1315	1226	2324
Klickitat	61	12	34	56	29	1259	336	0	33	132
Lewis	0	0	0	0	0	0	0	0	0	0
Lincoln	61	9	66	19	39	9	0	32	43	29
Mason	0	0	0	0	0	0	0	0	0	0
Okanogan	734	1798	1087	2470	1061	1109	1354	1176	396	214
Pacific	0	0	0	0	0	0	0	0	0	0
Pend Oreille	0	0	0	0	0	0	0	0	0	0
Pierce	0	0	0	0	0	0	0	0	0	0
San Juan	0	0	0	0	0	0	0	0	0	0
Skagit	0	0	0	0	0	0	0	0	0	0
Skamania	0	0	0	0	0	0	0	0	0	0
Snohomish	0	0	0	0	0	0	0	0	0	0
Spokane	83	107	0	52	73	23	35	79	9	48
Stevens	0	0	0	0	0	0	0	0	0	0
Thurston	0	0	0	0	0	0	0	0	0	0
Wahkiakum	0	0	0	0	0	0	0	0	0	0
Walla Walla	13	0	4	0	45	0	0	45	38	22
Whatcom	0	0	0	0	0	0	0	0	0	0
Whitman	420	195	240	266	593	84	690	143	155	128
Yakima	441	1877	1465	1520	3766	1852	735	1235	669	1114
