# Supplementary material for: A machine learning approach to managing game bird introductions
Source: PeerJ. 2025 Nov 4;13:e20291. doi: 10.7717/peerj.20291 (PMC12593725; doi:10.7717/peerj.20291)
Supplement: Supplemental Information 4 [file peerj-13-20291-s004.docx]

Supplementary Table S2:

**Hunting harvest statistics for Chukar in Washington State (2014–2023).**

| **Year** | **Unit 1** | **Unit2** | **Unit3** | **Unit4** | **Unit5** | **Unit6** | **Unit7** | **All** |
| --- | --- | --- | --- | --- | --- | --- | --- | --- |
| 2014 | 0 | 0 | 3364 | 2295 | 3807 | 82 | 23230 | 32778 |
| 2015 | 0 | 0 | 6243 | 2228 | 10251 | 353 | 14546 | 33621 |
| 2016 | 0 | 0 | 817 | 3191 | 8385 | 1367 | 17026 | 30786 |
| 2017 | 0 | 0 | 4279 | 3630 | 3907 | 224 | 11418 | 23458 |
| 2018 | 0 | 0 | 4425 | 3816 | 9339 | 183 | 14314 | 32077 |
| 2019 | 0 | 0 | 2157 | 1127 | 1836 | 789 | 16492 | 22401 |
| 2020 | 0 | 0 | 8921 | 2136 | 10071 | 990 | 11123 | 33241 |
| 2021 | 0 | 0 | 7248 | 6181 | 10400 | 2106 | 30926 | 56861 |
| 2022 | 0 | 0 | 8283 | 6577 | 14471 | 1717 | 33311 | 64359 |
| 2023 | 0 | 0 | 7522 | 4920 | 26513 | 1931 | 48409 | 89295 |
