# Supplementary figures and images for: A machine learning approach to managing game bird introductions
Source: PeerJ. 2025 Nov 4;13:e20291. doi: 10.7717/peerj.20291 (PMC12593725; doi:10.7717/peerj.20291)

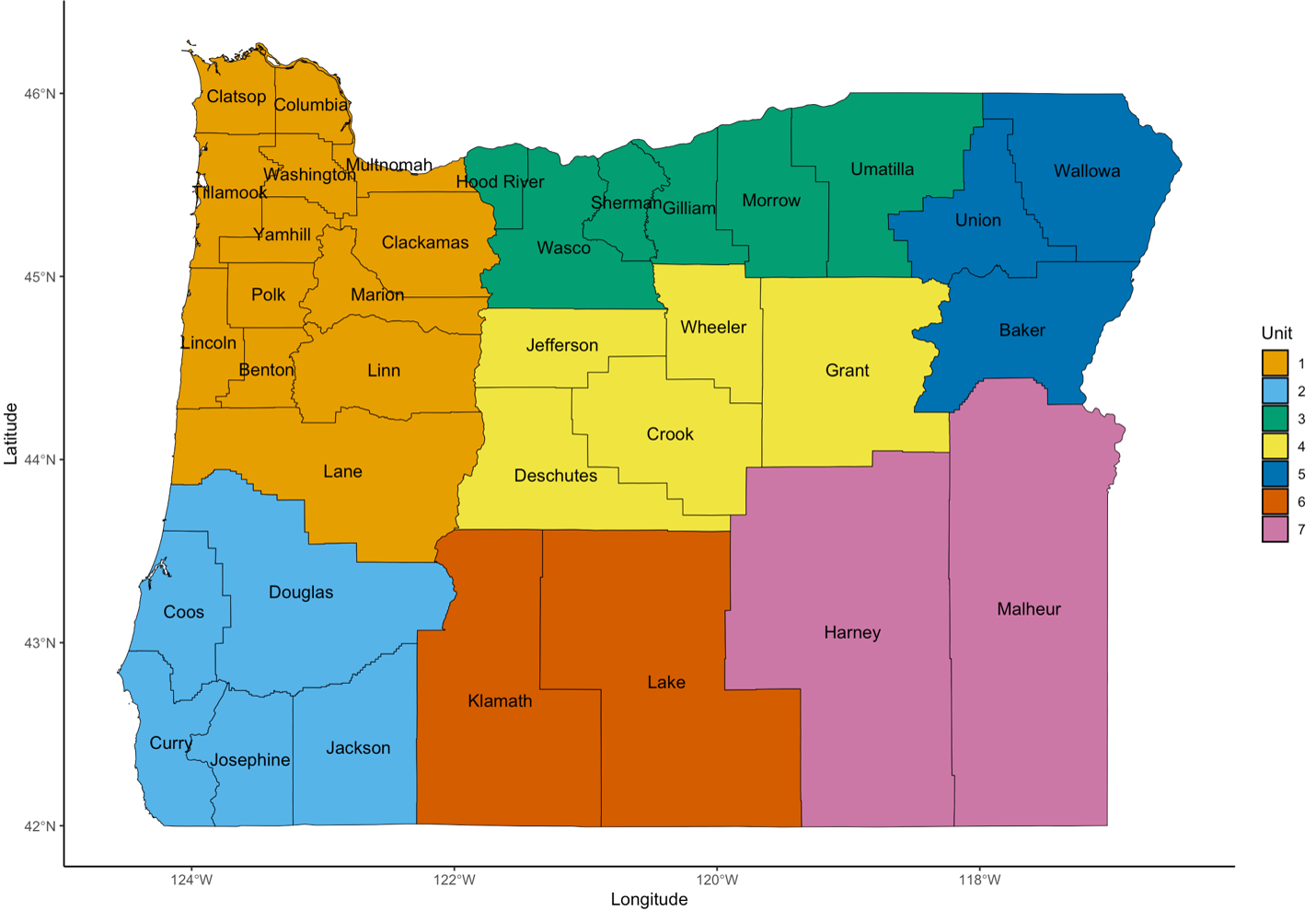

Supplement: Supplemental Information 5 — Maps were created by Austin M. Smith using R with Natural Earth polygon files (https://www.naturalearthdata.com), GBIF records (https://doi.org/10.15468/dl.5ybphp), and the records identifying the counties were based on Galbreath & Moreland (1953) and the Oregon Department of Fish and Wildlife. 2014–2023 (https://myodfw.com/articles/upland-birds-harvest-information). [file peerj-13-20291-s005.png]
